# Supplementary material for: Prevalence and factors associated with celiac disease in high-risk patients with functional gastrointestinal disorders
Source: PLoS One. 2024 Jun 7;19(6):e0297605. doi: 10.1371/journal.pone.0297605 (PMC11161043; doi:10.1371/journal.pone.0297605)
Supplement: Attachment — Submitted filename: Rebuttal Letter & Response to Editor - Reviewers.docx [file pone.0297605.s001.docx]

Rebuttal Letter

**Ari Fahrial Syam**

Division of Gastroenterology, Department of Internal Medicine, Faculty of Medicine

Universitas Indonesia/Dr. Ciptomangunkusumo Hospital, Jakarta, Indonesia

6th Salemba Raya Street, Senen District,

Central Jakarta City, Special Capital Region of Jakarta 10430

March 20, 2024

Dear Editor and Reviewers,

Beforehand, we would like to convey a heartfelt gratitude for your feedback and helpful comments for correction or modification on behalf of our submission [PONE-D-24-00819] and the opportunity to resubmit a revised copy of this manuscript.

We hope we have resulted in an improved revised manuscript, which you will find uploaded alongside this document. The manuscript has been revised to address the reviewer's comments, which are appended alongside our responses to this letter.

We hope the revised manuscript is accepted for publication in your journal.

Thank you for your consideration of this manuscript.

Sincerely,

**Ari Fahrial Syam**

**Response to Editor & Reviewers**

| No | Addressed Issue | Authors Response |
| --- | --- | --- |
| By Academic editor | | |
| 1 | Please ensure that your manuscript meets PLOS ONE's style requirements, including those for file naming. The PLOS ONE style templates can be found at  https://journals.plos.org/plosone/s/file?id=wjVg/PLOSOne_formatting_sample_main_body.pdf and  https://journals.plos.org/plosone/s/file?id=ba62/PLOSOne_formatting_sample_title_authors_affiliations.pdf | Thank you. We have applied your instruction on the revised manuscript. |
| 2 | funding information should not appear in the Acknowledgments section or other areas of your manuscript. We will only publish funding information present in the Funding Statement section of the online submission form.  Please remove any funding-related text from the manuscript and let us know how you would like to update your Funding Statement. | Thank you. We have removed funding information on our revised manuscript. |
| 3 | In the online submission form, you indicated that [Data cannot be shared publicly and are available upon request.].  All PLOS journals now require all data underlying the findings described in their manuscript to be freely available to other researchers, either 1. In a public repository, 2. Within the manuscript itself, or 3. Uploaded as supplementary information. This policy applies to all data except where public deposition would breach compliance with the protocol approved by your research ethics board. If your data cannot be made publicly available for ethical or legal reasons (e.g., public availability would compromise patient privacy), please explain your reasons on resubmission and your exemption request will be escalated for approval. | After careful consideration, we have agreed that our data remains and cannot be shared publicly and made available upon request. This is due to our commitment to our ethics board, which firmly believes data obtained upon conducting research is as private and confidential as a patient’s medical record. However, once it is needed for research/audit purposes we are ready to provide it as soon as possible. |
| 4 | When completing the data availability statement of the submission form, you indicated that you will make your data available on acceptance. We strongly recommend all authors decide on a data sharing plan before acceptance, as the process can be lengthy and hold up publication timelines. Please note that, though access restrictions are acceptable now, your entire data will need to be made freely accessible if your manuscript is accepted for publication. This policy applies to all data except where public deposition would breach compliance with the protocol approved by your research ethics board. If you are unable to adhere to our open data policy, please kindly revise your statement to explain your reasoning and we will seek the editor's input on an exemption. Please be assured that, once you have provided your new statement, the assessment of your exemption will not hold up the peer review process. | We are on the process of sorting and planning the data sharing. Once it is needed for research/audit purposes we are ready to provide it as soon as possible. |
| By Reviewer 1 | | |
| 1 | While the authors have attributed the reported increasing prevalence of CD to better tests availability as well as increasing gluten consumption, it must be clarified that the gross quantum of gluten consumption per person has not been implicated in the pathogenesis of CD -- it may be due to more persons consuming gluten. Besides, better tests availability and awareness are more important factors | Agree, thank you for your correction. We have corrected our previous statement in our revised manuscript. Related to test availability and patient awareness, we have mentioned it in discussion section, part: history of illness. |
| 2 | FGID and IBS are heterogeneous conditions, with patients presenting with a wide spectrum of symptoms. It would have been better if the authors selected either the general population or a subgroup with IBS-d. That would have made symptom analysis more meaningful | Exactly, however, our study focus is celiac disease and celiac clinical manifestations are not limited to IBS but also another kind of FGID such as functional diarrhea. We would like to capture celiac patients as variation as possible. |
| 3 | FGID / IBS by definition are low-risk conditions; the presence of high-risk features should warn against such diagnoses. Do the authors mean that these patients with FGID / IBS had other features (independent of FGID / IBS) that placed them at higher possibility of a diagnosis of CD? That is reasonable -- in the final analysis, only autoimmune disease correlated with the possibility of CD | That was our study focus, and this topic was also the first to be conducted in Indonesia. |
| 4 | Do the authors have an explanation why patients with CD were more likely to be aged 40-60 y rather than younger (which is expected)? | Studies of serial serum samples have reported loss of gluten tolerance as aging start on 35-40 years old. This is may be the cause CD serologic result are most prominent at age >40 years old in our study. However, CD can develop at any age; the earlier gluten exposure start, the earlier CD develop. There are differences in clinical manifestation between children and adult CD patients. In children, most CD manifest as diarrhea, weight loss and growth impairment. While in adults, most CD manifest as constipation, steatorrhea, obesity and micronutrients deficiency.  Thank you for the question, we have added this topic on discussion section of revised manuscript. |
| By Reviewer 2 | | |
| 1 | According to ESPGHAN updated guidelines, IgA anti-tissue transglutaminase (anti-TTG) is recommended but IgG anti-deaminated gliadin peptide (antiDGP) is not recommended. Why did the authors use IgG anti-deaminated gliadin peptide (antiDGP) serologic test in high risk population for celiac disease? Please explain it. | We remain to use anti-DGP in our study as anti-DGP has proven to has higher specificity than anti-TTG towards CD, while anti-TTG has higher sensitivity than anti-DGP. The sensitivity, specificity, and diagnostic accuracy of the IgA anti-tTG test were 86.3%, 50.0%, and 68.6%, respectively, and those of the IgG anti-DGP test were 95.4%, 85.7%, and 90.7%, respectively. Although, in any circumstances, the gold standard examination for CD remain biopsy of duodenum with suggestive finding: villous atrophy.  References:   - Anbardar, M. H., Haghighi, F. G., Honar, N., & Zahmatkeshan, M. (2022). Diagnostic Value of Immunoglobulin G Anti-Deamidated Gliadin Peptide Antibody for Diagnosis of Pediatric Celiac Disease: A Study from Shiraz, Iran. *Pediatric gastroenterology, hepatology & nutrition*, *25*(4), 312–320. <https://doi.org/10.5223/pghn.2022.25.4.312> - Jafari, S. A., Alami, A., Sedghi, N., Kianifar, H., Kiani, M. A., Khalesi, M., & Derafshi, R. (2023). Diagnostic accuracy of anti-DGP (IgG) for celiac disease. *Journal of family medicine and primary care*, *12*(1), 42–46. https://doi.org/10.4103/jfmpc.jfmpc_326_22 |
| 2 | The section of the introduction and discussion is not well organized. The manuscript did not cite the recent published articles. If the recent published articles are cited and discussed in the manuscript, it would be better. There are many articles on this subject, the authors can benefit from them. | Thank you, we have reorganized introduction and discussion section, also added several more recent publication. |
| 3 | The manuscript appropriately cites the important and authoritative references but does not cite the recent published articles. If the recent published article about celiac disease for example ‘’ Celiac disease in children: A review of the literature. World J Clin Pediatr. 2021 Jul 9;10(4):53-71’’ are cited, the manuscript would be better. | Thank you for your suggestion. We have added several more recent publication. |
| 4 | In the current study, the authors did not cite the recent published articles. If the recent published articles about prevalence of CD in high risk population for example ‘Prevalence of celiac disease in children with type 1 DM in the South of Turkey. Iran J Pediatr 2020;30:e97306’’ and ‘’The frequency of celiac disease in children with autoimmune thyroiditis. Acta Gastroenterol Belg. 2018 Jan-Mar;81(1):5-8’’and ‘’The frequency of celiac disease in children with chronic constipation. Kuwait Medical Journal 2021; 53 (4): 383 – 387’’ are cited, the manuscript would be better. | Thank you for your suggestion. We have added several more recent publication. |
| 5 | If the recent published articles about HLA-DQ2 and DQ8 in celiac patients for example ‘’Frequency of celiac disease and distribution of HLA-DQ2/DQ8 haplotypes among siblings of children with celiac disease. World J Clin Pediatr 2022 Jul 9;11:351-359’’ are cited, the manuscript will be better. | Thank you for your suggestion. We have added several more recent publication. |
